# Shear Wave Velocity of the Thenar Muscle Is Associated With the Neurophysiologic Severity of Carpal Tunnel Syndrome

**DOI:** 10.1097/WNP.0000000000001157

**Published:** 2025-03-19

**Authors:** Henri Grönfors, Katri Mäkelä, Sari-Leena Himanen

**Affiliations:** * Faculty of Medicine and Health Technology, Tampere University, Tampere, Finland; and; † Department of Clinical Neurophysiology, Tampere University Hospital, Wellbeing Services County of Pirkanmaa, Tampere, Finland.

**Keywords:** Shear wave elastography, Abductor pollicis brevis, Electrodiagnostic studies, Electroneuromyography

## Abstract

**Purpose:**

Aim of the study was to examine the associations between abductor pollicis brevis (APB) muscle stiffness evaluated by shear wave elastography and electrodiagnostic study findings in patients with carpal tunnel syndrome. The association between shear wave elastography and APB muscle echogenicity was also examined.

**Methods:**

This prospective study included patients who were referred to electrodiagnostic studies because of upper limb symptoms. The electrodiagnostic studies consisted of nerve conduction studies and needle-electromyography. Abductor pollicis brevis muscle shear wave velocity was measured, and muscle echogenicity assessed using the Heckmatt grading scale.

**Results:**

In total, 97 hands were included in the nerve conduction studies. Of these, 53 APB muscles were further examined with needle-electromyography. Shear wave velocity correlated positively with the neurophysiologic severity of carpal tunnel syndrome (*r* = 0.449, *P* = 0.028, *N* = 26). Mean shear wave velocity was faster in the APB muscles with neurogenic findings (mean 2.72 m/second, ±SD 0.36) than muscles with normal findings (2.48 m/second, ±SD 0.34, *P* = 0.036). In receiver operating characteristic analysis, the best shear wave velocity cutoff value was 2.66 m/second. With this cutoff value, the sensitivity was 0.692, while the 1-specificity was 0.233. Only seven APB muscles showed increased echogenicity.

**Conclusions:**

Shear wave velocity of APB muscle is positively associated with the neurophysiologic severity of carpal tunnel syndrome. Carpal tunnel syndrome-related axonal damage also seems to increase shear wave velocity in APB muscle; however, according to the receiver operating characteristic analysis, the method is not yet suitable for clinical use to define muscle denervation. The findings of this study show that shear wave elastography has potential as an additional clinical tool in the diagnostics of carpal tunnel syndrome.

The diagnosis of carpal tunnel syndrome (CTS) is primarily based on clinical evaluation. Electrodiagnostic (EDX) studies can be used to verify the diagnosis and in deciding whether treatment should be managed surgically or conservatively.^1^ Also, the neurophysiologic severity of CTS can be classified according to EDX findings.^2,3^ Presence of CTS-related axonal damage is evaluated in needle-electromyography (EMG) of a median nerve (MN) innervated thenar muscle, such as the abductor pollicis brevis (APB). Axonal damage can also be indirectly deduced from reduced MN sensory nerve action potential (SNAP) and compound muscle action potential (CMAP) amplitudes in the nerve conduction studies (NCS).^4^ However, the amplitudes may also decrease because of temporal dispersion caused by nerve demyelination, even without axon loss.

After denervation, muscle tissue is replaced by fibrotic and adipose tissues.^5–8^ These changes can be seen in ultrasound (US) brightness mode by increased echogenicity when compared with healthy muscle.^9^ The conventional method to estimate the extent of muscle pathology with US is the semiquantitative Heckmatt scale (HS).^10^ In addition, quantitative muscle gray scale analysis has been introduced, although it is not yet widely used in everyday practice.^11,12^ Physiologic increase of fibrotic and adipose tissue, relating, for example, to aging or obesity, likewise increases muscle echogenicity, which complicates its usage in assessment of muscle denervation in practice. Also, US image brightness is not standardized between US device manufacturers, and it is reliant on the settings of the used US device, which limits the comparability of studies addressing the echointensity.

Shear wave elastography (SWE) is a relatively new US-based method to estimate tissue stiffness. In SWE, the US probe produces pressure changes in tissue that cause transverse mechanical waves called shear waves. The propagation velocity of the shear waves can then be measured quantitatively. Interestingly, changes in SWE measurements have been found to relate to the pathophysiology of muscle tissue.^13,14^ Changes in the elastic properties of injured muscle also relate to an increase in intramuscular fibrotic and adipose tissues.^15–17^

Because SWE is a noninvasive and a quantitative method, it might prove to be a useful additional tool in the diagnostics of CTS. The main aim of this study was to investigate whether stiffness of APB muscle changes in CTS by testing associations between NCS parameters and APB muscle SWE. Another aim was to determine whether SWE can be used to detect APB muscle denervation in CTS by testing associations between needle-EMG and SWE findings. Third aim was to study the association between APB muscle SWE and HS. In addition, we also tested the association between the HS and EDX parameters.

## MATERIALS AND METHODS

The study material of this prospective study consisted of patients referred for EDX study because of upper limb symptoms. After a routine EDX study, the patients volunteered for US and SWE studies. The studies were conducted at the Department of Clinical Neurophysiology at Tampere University Hospital during the fall of 2021. All patients gave a written informed consent, and the study was approved by the Ethical Committee of Tampere University Hospital (Permit no. R19002). Patients who had EDX findings indicating muscle disease, C5-T1 radiculopathy, or a bifid MN in US, were excluded from the study, because these conditions might influence muscle SWE, echogenicity, or EDX parameters. Patients with postoperative CTS were excluded from the NCS statistical analyses but included in needle-EMG statistics. Patient age, sex, weight, height, and body mass index (BMI) were recorded in addition to the EDX, US, and SWE study results.

The EDX devices used were the Cadwell Sierra Summit EDX Solution (Cadwell Industries Inc, Kennewick, WA) and the Dantec Keypoint EMG Workstation (Natus Medical Inc, San Carlos, CA). The EDX studies were performed by a clinical neurophysiologist or residents specializing in clinical neurophysiology. In NCS, the SNAP responses were studied antidromically in median (digits 2 to 4) and ulnar (digits 4 and 5) nerves. Compound muscle action potential responses were tested for the median and ulnar nerves and were recorded with surface electrodes. The presence of CTS and its neurophysiologic severity were assessed according to the scoring system by Padua et al.^2^ and the reference values of our laboratory. Median nerve SNAP and CMAP were used to categorize the NCS findings as follows: Mild CTS: MN SNAP conduction velocity ≥10 m/second slower than the ulnar and CMAP motor distal latency (MDL) <4.2 ms. Moderate CTS: MN MDL ≥4.2 ms in addition to slow SNAP velocity as in the mild category. Severe CTS: MN MDL ≥4.2 ms and absent MN SNAPs. Extreme CTS: lack of both MN SNAP and CMAP. For statistical analyses, the following MN parameters were documented from each studied hand: neurophysiologic CTS severity classification, index finger SNAP latency, index finger SNAP amplitude, CMAP amplitude, and MDL.

Needle-EMG was performed on several upper limb muscles from myotomes C5-T1 to rule out possible radiculopathy or other neuropathies that might have affected the findings. Furthermore, needle-EMG was used to observe neurogenic findings of the APB muscle. In needle-EMG, neurogenic findings can be detected by the appearance of spontaneous activity or large and/or polyphasic motor unit potentials with a decreased interference pattern.^18^

For statistical analyses, the presence of neurogenic lesion was recorded, and the interference pattern was estimated as normal or reduced. In cases of symmetrical symptoms, needle-EMG was only performed on the other arm.

The US device used was the Samsung RS85 Prestige with a LA2-14A linear transducer. The frequency of US was 13 MHz. The gain and depth were kept constant. The US studies were performed by a clinical neurophysiologist who had 4 years' experience of neuromuscular US at the time of the study. The US operator was blinded to the EDX findings.

The US studies of the APB muscle were performed while the patient was sitting in a relaxed position, with the hand laying naturally relaxed on a table and the elbow joint at an angle of approximately 90°. The palmar side of the hand was placed facing upward, and the fingers were in relaxed semiflexion. First, the direction of the APB muscle fibers was determined in B-mode and the HS was estimated from a transverse oriented image with a scale of 1 to 4 as follows: HS1: normal muscle echogenicity. HS2: increased muscle echogenicity with clear distinction of nearby bone. HS3: profound increase of muscle echogenicity and decreased distinction of nearby bone echo. HS4: intense muscle echogenicity and lack of distinction of bone echo.^10^

**FIG. 1.**
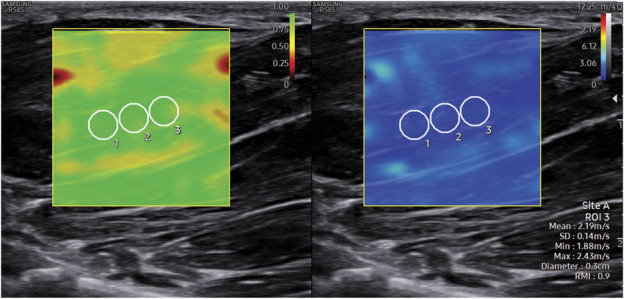
The APB muscle visualized in longitudinal orientation in a dual image. On the left, the overlaid color map shows the reliable measurement index. The SWE stiffness map is shown on the right. Three region of interest points are inserted on the muscle. The needle-EMG finding was normal in this muscle. The mean SWV of the three inserted region of interest points was 2.19 m/second. APB, abductor pollicis brevis; EMG, electromyography; SWE, shear wave elastography; SWV, shear wave velocity.

Thereafter, the transducer was rotated 90° and shear wave velocity (SWV) was measured by holding the transducer in a longitudinal position to the muscle fibers. Care was taken not to apply any pressure on the transducer. Three regions of interest were chosen approximately in the middle of the muscle, thereby avoiding clear intramuscular fibrous septae. A reliability measurement index was displayed simultaneously on the SWE stiffness map as a dual image. The reliability measurement index scale is from 0 to 1, with 1 representing the most reliable measurement. Although the manufacturer of the US device states that a reliability measurement index of 0.4 is sufficient to achieve reliable measurements, the regions of interest were placed in an area where the reliability measurement index was as close to 1 as possible, but at least 0.7. Thereafter, mean SWV from the three regions of interest was calculated. For statistical analyses, the SWV measurements were recorded in meters per second (m/second). The diameter of a region of interest was 3 mm. The SWE method is demonstrated in Fig. 1.

Statistical analyses were performed using IBM SPSS Statistics 29.0. We used the following statistical tests: one-way ANOVA, Mann–Whitney *U*-test, χ^2^ test, and Spearman correlation when appropriate. A receiver operating characteristic analysis was also performed. For statistical analyses, the severe and extreme neurophysiologic CTS severity groups were combined. Furthermore, hands that showed normal NCS results formed the “NCS-negative” group and the hands that had findings in the NCS study indicating CTS formed a “NCS-positive” group. To study the effect of patient age on the findings, the patients were divided into two age groups: younger than 60 years and 60 years and older.

## RESULTS

In total, 56 patients, 19 men and 37 women, volunteered for the study. Of these, 36 patients were younger than 60 years (64 hands) and 20 who were aged 60 years and older (33 hands). Patient characteristics are presented in Table 1. From the 56 patients, 97 hands were studied in the NCSs. Eight hands were postoperative and were excluded from the NCS statistical analyses. Of the 89 hands, NCS was normal in 63 hands (NCS-negative group), while 26 hands showed findings indicating CTS (NCS-positive group). The 26 hands in the NCS-positive group were further classified into mild (*N* = 11), moderate (*N* = 11), and severe-to-extreme CTS groups (*N* = 4).

**TABLE 1.** Mean, Range, and SD of Patient Age, Weight, Height, and BMI

	N	Minimum	Maximum	Mean	SD	Variance
Age (years)	56	30	86	54	± 16.1	249
Weight (kg)	56	50	128	77.4	± 19.1	356
Height (cm)	56	152	187	168.8	± 8.1	65
BMI (kg/m^2^)	56	18	41	28	± 6.3	40

BMI, body mass index.

In the NCS-positive group, SWV correlated positively to neurophysiologic severity (*r* = 0.449 *P* = 0.028). Furthermore, regarding the individual NCS parameters, SWV correlated positively with the MDL (*r* = 0.551, *P* = 0.004), SNAP latency (*r* = 0.580, *P* = 0.004), but negatively with SNAP amplitude (*r* = −0.411, *P* = 0.037). Moreover, SWV did not correlate with CMAP amplitude when all patients were included in the analysis. There was a significant negative correlation with CMAP amplitude and SWV among patients who were younger than 60 years of age (*r* = −0.560, *P* = 0.046). The negative association between the SWV and the SNAP amplitude strengthened when studied in the subgroup of patients who were younger than 60 years of age (*r* = −0.758, *P* = 0.003). In the NCS-negative group, the SWV did not correlate with index finger SNAP latency, SNAP amplitude, MDL, or CMAP amplitude.

**FIG. 2.**
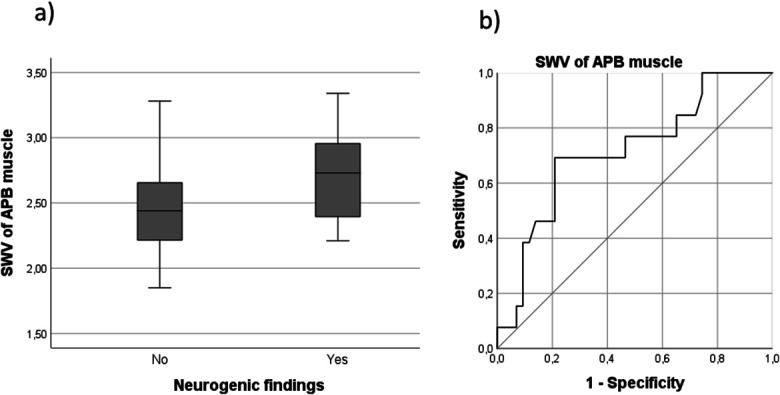
**A**, SWV (m/second) in the APB muscle in groups with and without neurogenic findings. **B**, ROC curve for SWV to discriminate the groups. APB, abductor pollicis brevis; ROC, receiver operating characteristic; SWV, shear wave velocity.

Needle-EMG was performed on the APB muscles of 53 hands. Of these, eight had undergone an MN release operation because of previously diagnosed CTS. The APB muscles of seven hands showed neurogenic findings (three in moderate and four in severe-to-extreme CTS groups). Shear wave velocity was significantly faster among the APB muscles that showed neurogenic findings in needle-EMG (mean 2.72 m/second, SD ± 0.36, 95% confidence interval for mean 2.52–2.93 m/second) compared with those muscles that did not (mean 2.48 m/second, SD ± 0.34, 95% confidence interval for mean 2.38–2.59 m/second) (*P* = 0.036). To discriminate these groups, a receiver operating characteristic analysis was performed to calculate the optimal SWV cutoff value. Area under the curve was 0.716 (95% confidence interval 0.554–0.877). The SWV cutoff value that optimized both sensitivity and specificity was 2.66 m/second. With this cutoff value, the sensitivity was 0.692 and 1-specificity 0.233. These findings are presented in Fig. 2.

**FIG. 3.**
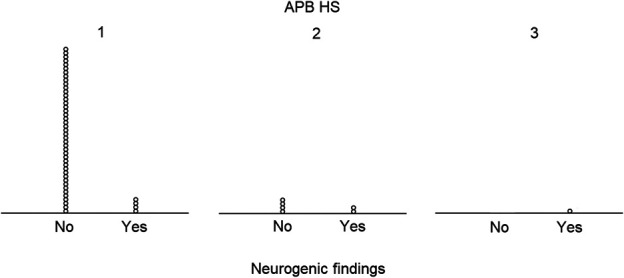
Distribution of the HS in relation to needle-EMG findings. EMG, electromyography; HS, Heckmatt scale.

As expected, all hands in the NCS-negative group had normal APB muscle echogenicity and were classified to the HS1 group in the Heckmatt grading scale assessment. Only seven hands showed increased echogenicity and were classified in HS2 (*N* = 6) and HS3 (*N* = 1) groups. There were statistically significantly neurogenic findings more often in the HS2 and HS3 groups than in the HS1 group (*P* = 0.001). The distribution of the HS groups according to the needle-EMG findings is presented in Fig. 3. All patients who showed increased echogenicity in the APB muscle were older than 60 years.

No significant difference in SWV was observed between the different HS groups. Furthermore, there was no significant difference in the distribution of the HS groups when the SWV was under or above the 2.66 m/second cutoff value.

Heckmatt scale correlated positively with patient age (*r* = 0.331, *P* = 0.001) and patient BMI (*r* = 0.229, *P* = 0.028). There was a nearly significant correlation with patient weight (*P* = 0.055), but no correlation with patient height was found. Shear wave velocity did not correlate with patient age, BMI, weight, or height. No correlations were found in the total material nor separately in the NCS-negative and NCS-positive subgroups.

## DISCUSSION

The results of this study show that the neurophysiologic severity of CTS and axonal damage related to CTS are associated with increased SWV in the APB muscle. The SWV of the APB muscle was associated with the NCS parameters that are traditionally used to determine the neurophysiologic severity of CTS with moderate correlations. Furthermore, the SWV in the APB muscle was significantly higher in muscles that showed neurogenic findings in needle-EMG than in intact muscles.

Previous research on the utility of SWE in the diagnostics of CTS has mainly focused on the SWV of the MN,^19–22^ whereas there is a scarcity of research on the SWE properties of MN innervated thenar muscles. One recent retrospective study has focused on this topic. In the study by Shin and coauthors,^23^ patients who had clinically suspected CTS underwent NCSs, but needle-EMG was not performed. Although the authors reported faster thenar muscle SWV in a CTS group than in a group of healthy controls, they found a weak negative correlation between CTS severity and thenar muscle SWV. In our study, there was a modest positive correlation between CTS severity and APB muscle SWV. In their discussion, Shin and coauthors speculate that the increased SWV of the thenar muscle in CTS could be due to denervation edema. Although the results of our current study confirm that increased SWV is associated with neurogenic findings, the presence of edema would need to be verified using other methods. Also a few other studies have studied the SWE of denervated muscles. For example, Alis and coauthors studied SWV in the multifidus muscle after lumbar disk herniation.^24^ Contrary to our findings, they found decreased SWV in affected multifidus muscles when compared with the healthy side. However, in their study, the US probe was kept transverse to the muscle fibers. It is commonly known that probe position, among several other variables, affects the acquired SWV values.^25^

Regarding the needle-EMG, although SWV was significantly faster among the APB muscles with neurogenic findings than intact muscles, the SWV values overlapped between the groups. Furthermore, receiver operating characteristic analysis indicates that the test is not suitable for clinical use. Previous findings suggest that the time the SWE study is performed in relation to the age of denervation may modify the results. In an animal model, muscle SWV was first found to decrease and then increase after denervation.^26^ In a study by Rosskopf and coauthors, the acquired SWV values were found to change nonlinearly in relation to the deterioration of the muscle pathology.^17^ This can be explained by the pathophysiologic process that denervated muscles undergo, where the muscle tissue is replaced by fibrous and adipose tissue sequentially. Increased fibrosis in muscles is associated with greater SWV compared with healthy muscle,^27^ whereas increased adipose tissue is associated with decreased SWV compared with healthy muscle.^17^ These dynamic changes limit the usability of SWE in clinical practice and may cause bias in research studies. In conditions such as CTS, however, the progression of the condition is usually typical and the behavior of SWV in relation to the time span of the condition could possibly be studied. In contrast, atypical rapid worsening of symptoms and edema may occur also in chronic phase. In future studies, interpretation of the SWE results in relation to the clinical findings and duration of symptoms could help move SWE toward clinical applicability.

Unfortunately, the number of APB muscles that had increased echogenicity turned out to be limited. There was, however, a positive correlation between echogenicity measuring Heckmatt grading scale (HS) and patient age as well as HS and BMI. It has been reported in the literature that patient age and BMI are associated with increased muscle echogenicity.^12,28,29^ Although there were statistically significantly neurogenic findings more often in muscles categorized as HS2 and HS3 (increased echogenicity) than in muscles categorized as HS1 (normal echogenicity), there were cases categorized as HS2 where needle-EMG was normal. It can be speculated, therefore, whether the echogenicity in these cases was increased physiologically in relation to patient age. Indeed, all patients who had increased echogenicity were older than 60 years. There were also cases that had neurogenic findings in needle-EMG but were classified as HS1. In conclusion, the presence of axonal damage related to CTS cannot be unequivocally deduced from the echogenicity of the APB muscle. Thus, it can be speculated that SWE could bring additional value to US-based evaluation of muscle tissue in future. In this study, SWV did not correlate with patient age, BMI, weight, or height. Previous studies have found contradictory results on associations of SWE with patient age, sex, and body composition.^23,30,31^ Thus this seems to be a subject for future studies to verify.

This study has also limitations. The main limitation is the small sample size. The number of APB muscles that showed neurogenic findings in needle-EMG or increased echogenicity was few. Because axonal damage is not common in CTS, where the primary pathophysiologic event is nerve demyelination, the results should be verified in a much larger study material. Furthermore, the time of CTS symptoms was not collected, which may cause bias because, as discussed earlier, muscle pathophysiologic processes are time related. Although the patients were asked to relax, the lack of surface-EMG leaves the possibility of slight muscle tension during SWE. Muscle contraction may increase SWV compared with a relaxed muscle.^30,32–34^ Also, the most sensitive tests for CTS in the NCS, such as the palm–wrist segment or short segment studies, were not performed, which leaves a possibility that very mild cases of CTS may have been classified into the NCS-negative group. Finally, HS is only a semiquantitative scaling system. Gray scale analysis would have been a more appropriate method to assess the echogenicity of muscle tissue and should be used in future research to study the associations between SWE and echogenicity.

Increased SWV in the APB muscle is associated with the neurophysiologic severity of CTS and related axonal damage. Although promising, APB muscle SWE is not yet suitable for clinical use for reliably detecting CTS or discerning or ruling out axonal damage. In future, further research on the associations between SWE, clinical findings, and duration of symptoms could provide a better understanding of the function of SWE in CTS and further enhance its clinical applicability.

## References

[1] Huisstede BM Fridén J Coert JH Hoogvliet P, European HANDGUIDE Group. Carpal tunnel syndrome: hand surgeons, hand therapists, and physical medicine and rehabilitation physicians agree on a multidisciplinary treatment guideline–results from the European HANDGUIDE Study. Arch Phys Med Rehabil 2014;95:2253–2263. 25127999 10.1016/j.apmr.2014.06.022

[2] Padua L LoMonaco M Gregori B Valente EM Padua R Tonali P. Neurophysiological classification and sensitivity in 500 carpal tunnel syndrome hands. Acta Neurol Scand 1997;96:211–217. 9325471 10.1111/j.1600-0404.1997.tb00271.x

[3] Werner RA Andary M. Carpal tunnel syndrome: pathophysiology and clinical neurophysiology. Clin Neurophysiol 2002;113:1373–1381. 12169318 10.1016/s1388-2457(02)00169-4

[4] Deng X Chau LH Chiu SY Leung KP Li SW Ip WY. Exploratory use of ultrasound to determine whether demyelination following carpal tunnel syndrome co-exists with axonal degeneration. Neural Regen Res 2018;13:317–323. 29557383 10.4103/1673-5374.226402 PMC5879905

[5] Ehmsen J Höke A. Cellular and molecular features of neurogenic skeletal muscle atrophy. Exp Neurol 2020;331:113379. 32533969 10.1016/j.expneurol.2020.113379

[6] Gonzalez D Contreras O Rebolledo D Espinoza J van Zundert B Brandan E. ALS skeletal muscle shows enhanced TGF-β signaling, fibrosis and induction of fibro/adipogenic progenitor markers. PLoS One 2017;12:e0177649. 28520806 10.1371/journal.pone.0177649 PMC5433732

[7] Joe AWB Yi L Natarajan A Muscle injury activates resident fibro/adipogenic progenitors that facilitate myogenesis. Nat Cell Biol 2010;12:153–163. 20081841 10.1038/ncb2015 PMC4580288

[8] Tews DS Goebel HH Schneider I Gunkel A Stennert E Neiss WF. Morphology of experimentally denervated and reinnervated rat facial muscle. I. Histochemical and histological findings. Eur Arch Otorhinolaryngol 1994;251:36–40. 8179865 10.1007/BF00175955

[9] Heckmatt J Dubowitz V Leeman S. Detection of pathological change in dystrophic muscle with B-scan ultrasound imaging. Lancet 1980;1:1389–1390. 6104175 10.1016/s0140-6736(80)92656-2

[10] Heckmatt JZ Leeman S Dubowitz V. Ultrasound imaging in the diagnosis of muscle disease. J Pediatr 1982;101:656–660. 7131136 10.1016/s0022-3476(82)80286-2

[11] Pillen S van Alfen N. Skeletal muscle ultrasound. Neurol Res 2011;33:1016–1024. 22196753 10.1179/1743132811Y.0000000010

[12] Wijntjes J van Alfen N. Muscle ultrasound: present state and future opportunities. Muscle Nerve 2021;63:455–466. 33051891 10.1002/mus.27081 PMC8048972

[13] Creze M Nordez A Soubeyrand M Rocher L Maître X Bellin MF. Shear wave sonoelastography of skeletal muscle: basic principles, biomechanical concepts, clinical applications, and future perspectives. Skeletal Radiol 2018;47:457–471. 29224123 10.1007/s00256-017-2843-y

[14] Taljanovic MS Gimber LH Becker GW Shear-wave elastography: basic physics and musculoskeletal applications. Radiographics 2017;37:855–870. 28493799 10.1148/rg.2017160116 PMC5452887

[15] Gilbert F Klein D Weng AM Supraspinatus muscle elasticity measured with real time shear wave ultrasound elastography correlates with MRI spectroscopic measured amount of fatty degeneration. BMC Musculoskelet Disord 2017;18:549. 29282062 10.1186/s12891-017-1911-8 PMC5745767

[16] Yoshiko A Ando R Akima H. Passive muscle stiffness is correlated with the intramuscular adipose tissue in young individuals. Eur J Appl Physiol 2023;123:1081–1090. 36637509 10.1007/s00421-023-05137-z

[17] Rosskopf A Ehrmann C Buck F Gerber C Flück M Pfirrmann C. Quantitative shear-wave US elastography of the supraspinatus muscle: reliability of the method and relation to tendon integrity and muscle quality. Radiology 2016;278:465–474. 26540450 10.1148/radiol.2015150908

[18] Kimura J. Electrodiagnosis in diseases of nerve and muscle: Principles and practice. 2nd ed. Philadelphia: F.A. Davis, 1984.

[19] Cingoz M Kandemirli S Alis D Samanci C Kandemirli G Adatepe N. Evaluation of median nerve by shear wave elastography and diffusion tensor imaging in carpal tunnel syndrome. Eur J Radiol 2018;101:59–64. 29571802 10.1016/j.ejrad.2018.02.005

[20] Kantarci F Ustabasioglu F Delil S Median nerve stiffness measurement by shear wave elastography: a potential sonographic method in the diagnosis of carpal tunnel syndrome. Eur Radiol 2014;24:434–440. 24220753 10.1007/s00330-013-3023-7

[21] Lin CP Chen IJ Chang KV Wu WT Özçakar L. Utility of ultrasound elastography in evaluation of carpal tunnel syndrome: a systematic review and meta-analysis. Ultrasound Med Biol 2019;45:2855–2865. 31402226 10.1016/j.ultrasmedbio.2019.07.409

[22] Park E Hahn S Yi J Shin K Lee Y Lee HJ. Comparison of the diagnostic performance of strain elastography and shear wave elastography for the diagnosis of carpal tunnel syndrome. J Ultrasound Med 2021;40:1011–1021. 32852107 10.1002/jum.15478

[23] Shin KJ Yi J Hahn S. Shear-wave elastography evaluation of thenar muscle in carpal tunnel syndrome. J Clin Ultrasound 2023;51:510–517. 36201602 10.1002/jcu.23359

[24] Alis D Durmaz E Alis C Shear wave elastography of the lumbar multifidus muscle in patients with unilateral lumbar disk herniation. J Ultrasound Med 2019;38:1695–1703. 30426520 10.1002/jum.14854

[25] Cipriano KJ Wickstrom J Glicksman M A scoping review of methods used in musculoskeletal soft tissue and nerve shear wave elastography studies. Clin Neurophysiol 2022;140:181–195. 35659822 10.1016/j.clinph.2022.04.013 PMC9394639

[26] Wen J Wang Y Jiang W Quantitative evaluation of denervated muscle atrophy with shear wave ultrasound elastography and a comparison with the histopathologic parameters in an animal model. Ultrasound Med Biol 2018;44:458–466. 29174043 10.1016/j.ultrasmedbio.2017.08.1887

[27] Liu K Bhatia K Chu W He L Leung S Ahuja A. Shear wave elastography—a new quantitative assessment of post-irradiation neck fibrosis. Ultraschall Med 2015;36:348–354. 25171602 10.1055/s-0034-1366364

[28] Katzberg H Bril V Breiner A. Ultrasound in neuromuscular disorders. J Clin Neurophysiol 2016;33:80–85. 27035247 10.1097/WNP.0000000000000234

[29] Stock M Thompson B. Echo intensity as an indicator of skeletal muscle quality: applications, methodology, and future directions. Eur J Appl Physiol 2021;121:369–380. 33221942 10.1007/s00421-020-04556-6

[30] Eby S Cloud B Brandenburg JE Shear wave elastography of passive skeletal muscle stiffness: influences of sex and age throughout adulthood. Clin Biomech (Bristol) 2015;30:22–27. 25483294 10.1016/j.clinbiomech.2014.11.011 PMC4298479

[31] Varol U Valera-Calero J Fernández-de-Las-Peñas C Buffet-García J Plaza-Manzano G Navarro-Santana MJ. Body composition and demographic features do not affect the diagnostic accuracy of shear wave elastography. Bioengineering (Basel) 2023;10:904. 37627789 10.3390/bioengineering10080904 PMC10451656

[32] Brandenburg J Eby S Song P Feasibility and reliability of quantifying passive muscle stiffness in young children by using shear wave ultrasound elastography. J Ultrasound Med 2015;34:663–670. 25792582 10.7863/ultra.34.4.663 PMC4369795

[33] Eby S Song P Chen S Chen Q Greenleaf J An KN. Validation of shear wave elastography in skeletal muscle. J Biomech 2013;46:2381–2387. 23953670 10.1016/j.jbiomech.2013.07.033 PMC3818126

[34] Shinohara M Sabra K Gennisson JL Fink M Tanter M. Real-time visualization of muscle stiffness distribution with ultrasound shear wave imaging during muscle contraction. Muscle Nerve 2010;42:438–441. 20665510 10.1002/mus.21723

